# On Some Urinary Tests I

**Published:** 1892-01-16

**Authors:** 


### ON SOME URINARY TESTS.—I.

From the very earliest times the importance of an examination of the renal secretion was regarded as important. To the Greek writers, with their scanty knowledge of anatomy and chemistry, it was an important indication of internal changes, which they had hardly any other method of getting at. In later times this was carried to such an excess that a diagnosis was made by inspection of the water even without seeing the patient. Shakespeare laughs at the popular superstition in the guarded report of Falstaff's physician when water was carried to him for his opinion, that it was a good healthy water, but as for the patient that owned it he might have more diseases than he knew for. With the investigation of Bright's disease, and the use of chemical analysis, new importance was gained for the practice, but we are very far from being able to analyse the secretion with accuracy, or to interpret the value of the results which we get. For instance the detection of small amounts of albumen is neither easy, nor are we always agreed as to its significance.

*Urea*, to commence with, which is excreted to the amount of about an ounce daily, that is, more than any other solid constituent of the urine, is comparatively little utilized as a test of physiological changes. It is increased in febrile diseases, congestion of the liver and diabetes, but diminished in some forms of kidney and wasting diseases, notably in cancer, hepatic abscess, diabetic coma, and acute gout. Liebig's method of testing with mercuric nitrate, and the methods by hypobromite or hypochlorate solutions are still those practically employed. While the first is the most accurate, those which depend on the evolution of nitrogen gas are the most rapid. The difficulty with simple apparatus is to reckon for the differences due to changes of temperature and pressure. Otherwise the plain bent tube of Doremus, graduated by Messrs. Southall, to show grains per ounce and millegrammes per centimetre merely requires filling with hypobromite solution or the liquor sodæ chlorinatæ (U.S.P.), and the introduction of a centimetre of urine taken from the whole excretion of twenty-four hours to give a fairly accurate result. Nothing can be quicker or easier to manage. Nor are the ureometers, which allow for difference of pressure, troublesome to work. The liquor sodæ chlorinatæ, though slower and less energetic, is probably preferable for frequent testing, as the hypobromite solution will not keep for more than a month, and the bromine is unpleasant to manipulate separately unless kept in capsules.

*Uric acid.* A great deal of interest has been excited about the estimation of uric acid, and the source whence it is derived, though only some seven grains a day are normally excreted. Some authorities go so far as to say that even this is merely the product of the kidney, while that produced in other parts of the body is oxydised, and never appears as such in the urine. Garrod, however, has shown that it occurs in greatly increased quantities in the body during gout, while its excretion is diminished, and Haigh endeavours to prove that by insufficient excretion (for as to increased production we have no evidence) and the consequent accumulation of uric acid in the system, we get not only gout, but high tension pulse, acute rheumatism, migraine, mental depression, and sometimes epilepsy. Alkalies and salicylates increase the excretion in the form of urates, while iron, lead, the mineral acids, opium, and morphia reduce it. Ralfe lays down that uric acid is eliminated in excess chiefly in diseases of the liver, such as acute yellow atrophy, cirrhosis and cancer, diseases of the spleen, and scurvy, or as a condition antecedent to the outbreak of cancer, phthisis, and diabetes, or the appearance of syphilis and gout. As the solubility of uric acid in the urine depends largely on the salines present, Johnson and Shearer explain the frequency of uric acid calculi in India, and Southern China by the scarcity of salt in the diet of those countries. Whether the excretion can be rendered freer by copious imbibition of water there is some conflict of evidence, but probably the solubility is greatest when the water is neither strongly acid nor alkaline, and is of low specific gravity. When the excretion of sugar in diabetes is stopped, the lessened flow of water is sometimes marked by the appearance of uric acid crystals. It makes all the difference to a patient whether free uric acid is painfully passed as such, or only crystallises out after standing for some time, but in neither case does it necessarily imply an excessive excretion any more than the common pink urates in concentrated urine do. The estimation of uric acid simply and quickly is still a desideratum. Arthaud and Butte devised a method by which sulpho-cyanide of copper in a solution of hyposulphite of sodium was converted into an insoluble urate. Haycraft's silver process is practically the best and quickest. Salkowski, however, points out that the precipitate obtained does not contain an entirely constant percentage of uric acid. His own method proceeds by precipitating the urate with ammoniacal silver solution, which is decomposed by sodium sulphide and the resulting urate of soda changed to uric acid by reaction with hydrochloric. In Haycraft's the ammoniacal silver solution is added after the precipitation of the phosphates. The urate of silver is collected on an asbestos filter, thoroughly washed, and the silver estimated by Volhard's method. After adding a few drops of potash alum a centinormal solution of sulphocyanate of ammonia is dropped in till a permanent red colour appears. One cubic centimetre of the sulpho-cyanate = ·00168 grammes uric acid. For rough estimation the plan of concentrating with the addition of acid, and then weighing the crystals formed may be used, but it is almost as lengthy as Haycraft's, and probably far less exact.

